# The Use of Clasps—Bonwill Method

**Published:** 1893-05

**Authors:** S. B. Palmer

**Affiliations:** Syracuse, N. Y.


### THE USE OF CLASPS—BONWILL METHOD.

BY S. B. PALMER, M.D.S., SYRACUSE, N. Y.

The February number of the International Dental Journal contains a paper read before the Odontological Society of Pennsylvania, June 7, 1890. I do not write to criticise the paper, but the author is in fault in having hid so much "light under a bushel" for more than two years. The title of the paper is "New Method of Clasped Plates *versus* Movable or Unmovable Bridge-Work." I regard the contribution the most practical, beneficial, and scientific of any I have read on prosthetic dentistry. Every operation is so well illustrated that any one versed in the working of gold can produce the results claimed.

The writer of the paper, Dr. W. G. A. Bonwill, includes the word "*versus*" in the title, which might lead some to conclude that the given method was the best, but conditions and circumstances must be considered in each case. I will not detract from Dr. Bonwill's contribution by claiming to have used his method before him, but will add some personal experience in the wear of clasp plates and unmovable bridges, which would give support to either extreme and in the durability or destructibility of clasp plates. I have witnessed in my own practice such varied results that I will venture to offer some well-known causes which will invariably produce corresponding results. First, to predict failure: Use thin elastic clasps well-fitted to the tooth, as wide as can be worn and without caps or tip, to prevent sliding up or down in mastication. The above conditions will bring about unfavorable results or loss of the tooth in from two to six years. The sliding motion wears the tooth-substance, the retention of food by decomposition and chemical action destroys the tooth much faster and deeper than is done by mechanical abrasion, while the closing up the clasp upon the tapering crown forces the plate into the gums and the tooth out of the socket where there is no antagonizing tooth. So much from observation in practice. The following is personal experience on the other extreme:

In 1848, forty-five years ago, the first operation or piece of dentistry I ever did was to insert for myself an upper silver plate of nine teeth with clasps upon a molar on either side, with no other instruction than Goddard's work, "The Human Teeth," bought for the purpose. Subsequently the silver plate gave place to gold, with another tooth added; later, rubber was tried, then swaged aluminum with rubber attachments; from that to cast aluminum, and back again to swaged aluminum. During forty-three years those molars were clasped with metal except a short time when rubber was used. One year ago one of the clasped teeth became loose from absorption of the process, the buccal roots became exposed, and the tooth was extracted; the crown was in good condition, enamel not worn through, and no decay. The other is firm and promises well for years to come.

What are we to learn from this extreme case of plate-support? First, that the teeth received quite as much support for their preservation from clasps as they in turn gave to the plate. The clasps were never wide, and only for a few years were they allowed to slide upon the teeth. When the teeth began to elongate, tips or caps rested upon the crowns which held the teeth firm in the sockets and the plate from sliding, which prevented wear as well as depression of the gums under the plate. Five years ago bridges were inserted below, which called for a cap over the ends of the molars to secure articulation, in which some knowledge has been gained respecting removable caps. To return to the bearing of clasps, follow the suggestions and illustrations; let the clasps be narrow and bear upon the crown in three or four places only, and always have a tip resting upon some depression in the enamel upon the grinding surface. Avoid the drilled depression if possible. The majority of patients require automatic cleansing principles inserted with their work. What cannot be done in the construction of the piece cannot be depended upon by instructing the patient. Thus the spaces are beneficial except at the points of contact. The caps mentioned should not turn over upon the crown of the tooth so much as to touch the clasp and thus make a retaining place for food. I found it advisable to drill a hole through the cap upon the grinding surface which gives circulation and thus prevents decomposition of food lodgements. The change which food undergoes when confined around the teeth by any means for more than five or six hours will be noticed by any one giving the mouth proper attention.

A few words in regard to bridges. I could speak in quite as positive terms on the benefits of bridges on the lower jaw as has been done for clasps upon the upper. The bridge-worker needs to know what can be accomplished by clasps upon the principle described by Dr. Bonwill, to avoid the sacrifice of sound teeth, which becomes necessary when only such teeth are available.

We read of torture inflicted by savages in skinning their victims alive. Limited to a tooth, the operation of shortening and grinding up to receive a properly-fitting band is not less barbarous, nor can it be said that clasps are more destructive than this treatment.

